# Metformin Suppresses Diethylnitrosamine-Induced Liver Tumorigenesis in Obese and Diabetic C57BL/KsJ-+Lepr^*db*^/+Lepr^*db*^ Mice

**DOI:** 10.1371/journal.pone.0124081

**Published:** 2015-04-16

**Authors:** Tomohiko Ohno, Masahito Shimizu, Yohei Shirakami, Atsushi Baba, Takahiro Kochi, Masaya Kubota, Hisashi Tsurumi, Takuji Tanaka, Hisataka Moriwaki

**Affiliations:** 1 Department of Gastroenterology/Internal Medicine, Gifu University Graduate School of Medicine, Gifu, Japan; 2 Department of Tumor Pathology, Gifu University Graduate School of Medicine, Gifu, Japan; University of Cordoba, SPAIN

## Abstract

Obesity and related metabolic disorders, such as diabetes mellitus, raise the risk of liver carcinogenesis. Metformin, which is widely used in the treatment of diabetes, ameliorates insulin sensitivity. Metformin is also thought to have antineoplastic activities and to reduce cancer risk. The present study examined the preventive effect of metformin on the development of diethylnitrosamine (DEN)-induced liver tumorigenesis in C57BL/KsJ-+Lepr*^db^/*+Lepr^*db*^ (*db/db*) obese and diabetic mice. The mice were given a single injection of DEN at 2 weeks of age and subsequently received drinking water containing metformin for 20 weeks. Metformin administration significantly reduced the multiplicity of hepatic premalignant lesions and inhibited liver cell neoplasms. Metformin also markedly decreased serum levels of insulin and reduced insulin resistance, and inhibited phosphorylation of Akt, mammalian target of rapamycin (mTOR), and p70S6 in the liver. Furthermore, serum levels of leptin were decreased, while those of adiponectin were increased by metformin. These findings suggest that metformin prevents liver tumorigenesis by ameliorating insulin sensitivity, inhibiting the activation of Akt/mTOR/p70S6 signaling, and improving adipokine imbalance. Therefore, metformin may be a potent candidate for chemoprevention of liver tumorigenesis in patients with obesity or diabetes.

## Introduction

Diabetes mellitus, a major complication of obesity, is a serious healthcare problem worldwide due to its high prevalence; the International Diabetes Federation estimated in 2008 that 246 million adults worldwide had diabetes and that the prevalence was expected to reach 380 million in 2025 [1]. Many diabetic patients die of cardiovascular and cerebrovascular events and infectious diseases. Cancer is also a major cause of death in patients with diabetes as obesity and diabetes significantly raise the risk of several types of malignancies, especially hepatocellular carcinoma (HCC) [2–5].

HCC is one of the most frequently occurring cancers worldwide, causing 0.7 million deaths each year [6]. Several pathophysiological mechanisms linking obesity, diabetes, and liver carcinogenesis have been demonstrated, including the development of insulin resistance and adipokine imbalance [7–9]. Hyperinsulinemia may directly contribute to the development of HCC because insulin, which plays a key role in the oncogenesis signal transduction network [10], induces HCC cells to proliferate and resist apoptosis [11–13]. Activation of the phosphatidylinositol 3-kinase (PI3K)/Akt/mammalian target of rapamycin (mTOR) pathway induced by unrestrained insulin is also associated with hepatocarcinogenesis [14]. Excessive activation of the Akt/mTOR pathway has a crucial role in obesity-associated insulin resistance [15], and is critically important in liver carcinogenesis [14, 16]. In addition, increased levels of leptin, but decreased levels of adiponectin, are involved in the progression of steatosis and related liver tumorigenesis [17–20]. These findings suggest that targeting insulin resistance and adipokine imbalances such as hyperleptinemia and hypoadiponectinemia might be an effective strategy for preventing obesity- and diabetes-related liver carcinogenesis [8, 9].

Metformin is one of the most widely used drugs for the treatment of type 2 diabetes mellitus. The drug improves insulin sensitivity, especially in skeletal muscles, decreases hepatic gluconeogenesis, and inhibits glycogenolysis [21]. Furthermore, both clinical and experimental studies have demonstrated that metformin exerts beneficial effects on cancer prevention and treatment in various organs, including liver [22–25]. Several potential mechanisms have been proposed to explain the anticancer and cancer preventive properties of metformin. For instance, metformin exerts antineoplastic effects through the activation of the energy sensor AMP-activated protein kinase (AMPK) [26, 27]. Inhibition of the Akt/mTOR signaling pathway is also a critical mechanism for the suppression of cancer cell proliferation by metformin [28]. Moreover, metformin has direct effects on adipocyte and cardiac muscle, resulting in reduction of leptin secretion [29] and induction of adiponectin secretion [30].

In a recent study, administration of metformin prevented chemically-induced liver tumorigenesis in mice [22]. Metformin also inhibits proliferation of HCC-derived cells via induction of cell cycle arrest at G_0_/G_1_ phase [31], and treatment of the mouse HCC xenograft model with metformin shows suppressed tumor growth [32]. Another study demonstrated that metformin treatment up-regulates the expression of p21^CIP^ and p27^KIP^, but down-regulates cyclin D1 levels, which are shown both in HCC cell lines and in tumor xenograft tissues [33]. In addition, Saito and colleagues [34] reported using flow cytometric analysis that metformin treatment significantly reduces the number of tumor-initiating epithelial cell adhesion molecule (EpCAM)-positive HCC cells, which are considered as hepatic cancer stem cells.

These findings suggest that metformin might be a useful agent for preventing HCC development; however, detailed studies clarifying the chemopreventive effects of metformin on obesity- and diabetes-related liver tumorigenesis have not yet been conducted. In the present study, we examined the preventive effects of metformin on hepatic carcinogen diethylnitrosamine (DEN)-induced liver tumorigenesis in C57BL/KsJ-+Lepr^*db*^ */*+Lepr^*db*^ (*db/db*) mice, which exhibit obesity and diabetes, focusing on improvements in insulin resistance, inhibition of the Akt/mTOR signaling pathway, and amelioration of adipokine imbalance.

## Materials and Methods

### Animals and chemicals

Male and female C57BL/KsJ-m+*/*+Lepr^*db*^ *(+/db)* mice were obtained from Japan SLC (Shizuoka, Japan) and were housed in plastic cages (2–3 mice per cage) with free access to drinking filtered tap water and a pelleted basal diet CRF-1 (Oriental Yeast Co., Ltd., Tokyo, Japan) under controlled conditions of humidity (50±10%), light (12/12 h light/dark cycle) and temperature (23±2°C). Mice were maintained in the specific pathogen free (SPF) facility at Gifu University Life Science Research Center in accordance with Institutional Animal Care Guidelines. The protocol was approved by the Committee on the Ethics of Animal Experiments of Gifu University (the authorization number is 25–8). DEN was purchased from Sigma Chemical Co. (St. Louis, MO, USA) and dissolved in phosphate buffered saline to make 1% (w/v) solution, according to manufacturer’s instruction. Metformin was supplied by Dainippon Sumitomo Pharma Co. (Tokyo, Japan). The maximum solubility of metformin is 346 mg/ml in water.

### Experimental procedure

Newborn *db/db* mice were obtained by mating male and female *+/db* mice. Female *db/db* mice were planned to be randomly divided into the following 4 experimental and control groups: no treatment control group (group 1); metformin alone group (group 2); DEN alone group (group 3); and DEN plus metformin group (group 4). Some mice, which would be in groups 3 and 4, received a single intraperitoneal injection of DEN (25 mg/kg body weight) at 2 weeks of age. Originally, groups 1 and 2 would have 5–6 mice as DEN-free control groups and groups 3 and 4 would have 10–20 mice as DEN-injected groups. Finally, DEN-free 11 mice and DEN-injected 32 mice were separated as follows: group 1 (n = 6), group 2 (n = 5), group 3 (n = 19), and group 4 (n = 13). At 4 weeks of age, mice in groups 2 and 4 were given tap water containing metformin (300 mg/kg/day) until the end of the experiment. Although water consumption was not measured in this study, previous report showed that *db/db* mice drink about 30 ml/100g/day of water [35], and then mice were supplied with 1.0 mg/ml of metformin. This concentration of metformin was also established according to previous chemopreventive studies [22, 36]. Mice in groups 1 and 3 were given tap water without metformin. At 24 weeks of age (after 20 weeks of metformin treatment), all mice were sacrificed to analyze the development of liver neoplasms and preneoplastic lesions, foci of cellular alterations (FCA). The histological definition of FCA is indicated by Popp and Goldsworthy [37].

### Histopathological analysis

At sacrifice, livers were immediately removed and maximum sagittal sections of 3 lobes (left lateral lobe, left medial lobe, and right medial lobe) were used for histopathological examination. For all experimental groups, 4-m thick sections of formalin-fixed, paraffin-embedded livers were stained with hematoxylin and eosin (H&E) for histopathology. The presence of HCC, liver cell adenoma, and FCA was judged according to previously described criteria [38]. The multiplicity of FCA was assessed on a per unit area (cm^2^) basis [17]. The histological features of the livers were evaluated by using the non-alcoholic fatty liver disease (NAFLD) activity score (NAS) system [39].

### Protein extraction and western blot analysis

Total protein was extracted from non-tumorous areas of liver samples (30 mg) using 500 μl lysis buffer [50 mM Tris–HCl (pH 8.0), 150 mM NaCl, 0.1% sodium dodecyl sulfate (SDS), 0.5% deoxycholic acid, 1% NP-40] containing protease inhibitors (Protease Inhibitor Cocktail Set I; Calbiochem) and Mini-BeadBeater-1 (BioSpec Products). Proteins (20 μg/lane) were separated by SDS–polyacrylamide gel electrophoresis and transferred onto nylon membranes (Immobilon-P Transfer Membranes; Millipore). Immunoblots were performed using primary antibodies. Primary antibodies for Akt (#9272), phosphorylated Akt (p-Akt; Ser473, #9271), mTOR (#2972), phosphorylated mTOR (p-mTOR; Ser2448, #2971), AMPK-α (#2603), phosphorylated AMPK-α (p-AMPK-α; Thr172, #2535), p70S6 (#9202), phosphorylated p70S6 (p-p70S6; Thr389, #9205), STAT3 (#9132), phosphorylated STAT3 (p-STAT3; Tyr705, #9231), and glyceraldehyde-3-phosphate dehydrogenase (GAPDH, #2118) were obtained from Cell Signaling Technology (Beverly, MA, USA). GAPDH was served as a loading control. Membranes were incubated with an appropriate horseradish peroxidase-conjugated secondary antibody (GE Healthcare). Each membrane was developed using an enhanced chemiluminescent substrate for the detection of horseradish peroxidase (Thermo Scientific), followed by densitometric scanning using NIH image software version 1.45 [40].

### Clinical chemistry

Blood samples, which were collected from inferior vena cava at sacrifice after 6 hours of fasting, were used for chemical analyses. Serum concentrations of insulin, leptin, and high molecular weight adiponectin were determined by an enzyme immunoassay according to the manufacturer’s protocols (Shibayagi, Gunma, Japan). Serum levels of glucose were measured using a glucose CII-test kit (Wako, Osaka, Japan). Insulin resistance and insulin sensitivity were determined by calculating the homeostatic model assessment of insulin resistance (HOMA-IR) and the quantitative insulin sensitivity check index (QUICKI), respectively [7, 17]. HOMA-IR index was calculated according to the formula: HOMA-IR = [fasting glucose (mmol/l)]*[fasting insulin (μU/ml)]/22.5. QUICKI was determined as follows: QUICKI = 1 ⁄ [log(I0) + log(G0)], where I0 is the fasting insulin (μU/ml) and G0 is the fasting glucose (mg/dl), which correlates with the glucose clamp method [41].

### Statistical analysis

The results are presented as the means ± SD, and were analyzed using JMP software version 10 (SAS Institute, Cary, NC, USA). Differences among the 4 groups were analyzed by one-way ANOVA. When the ANOVA showed a statistically significant effect (*P* < 0.05), each experimental group was compared by Tukey-Kramer’s multiple comparisons test. The differences were considered significant at *P* values of less than 0.05.

## Results

### General observations

No significant differences were observed in body weights or relative weights of the livers and white adipose tissue (periorchis and retroperitoneum) among the 4 groups at the end of the experiment (Table 1). During the experiment, no clinical symptoms of metformin toxicity were seen. Histopathological examination also did not show metformin toxicity in important organs including the liver, kidney, and spleen.

**Table 1:** Body, liver, and fat weights of the experimental mice.

Group no.	Treatment	No. of mice	Body wt (g)	Relative wt (g/100 g body wt)	
				Liver	Fat^a^
1	None	6	58.2± 8.8^b^	6.6± 1.1	5.2± 0.8
2	metformin alone	5	56.0± 11	6.8± 0.5	5.7± 1.8
3	DEN alone	19	60.5± 11	6.9± 1.5	5.9± 1.6
4	DEN + metformin	13	59.0± 13	6.6± 1.4	6.0± 1.7

^a^White adipose tissue of the periorchis and retroperitoneum.

^b^Mean ± SD.

### Effects of metformin on DEN-induced liver tumorigenesis in *db/db* mice

**Fig 1:**
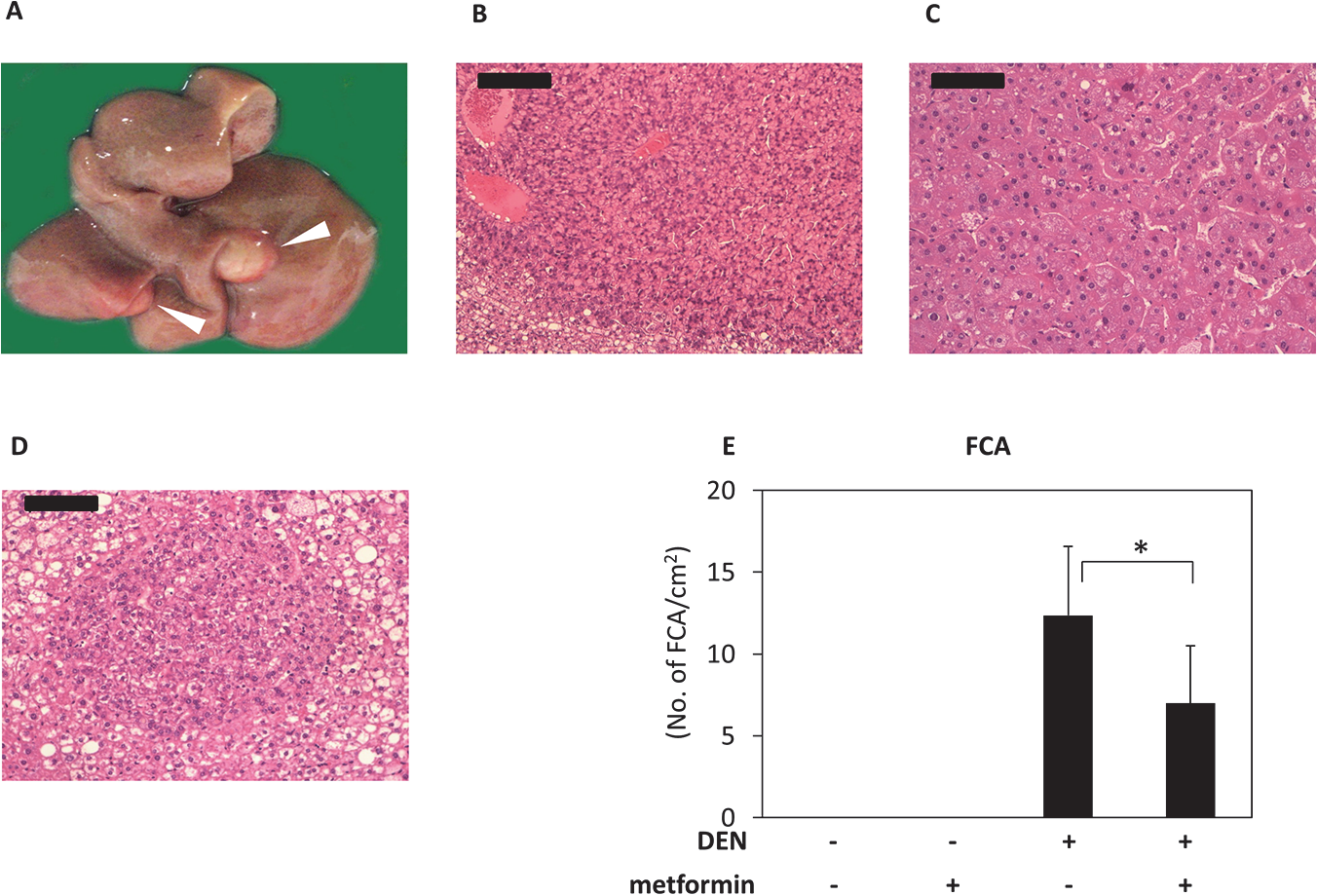
Macroscopic and microscopic analyses of liver neoplasms in DEN-treated *db/db* mice and effects of metformin on DEN-induced FCA. (A) The whitish and nodular tumors (HCC; arrowheads) are present in the liver of DEN-treated *db/db* mouse. (B, C, and D) Paraffin-embedded sections stained with H&E. Representative histopathology of (B) liver cell adenoma, (C) HCC, and (D) FCA. Bars are (B) 200 μm and (C and D) 100 μm. (E) Average numbers of FCA in all groups [untreated control group (n = 6), metformin alone group (n = 5), DEN alone group (n = 19), and DEN plus metformin group (n = 13)]. Each column represents the mean ± SD. * *P* < 0.001.

Macroscopically, whitish and nodular tumors were observed only in the livers of mice treated with DEN alone group at the end of the study (Fig 1A). As listed in Table 2, hepatic neoplasms, including liver cell adenoma (Fig 1B) and HCC (Fig 1C), developed only in the livers of mice that received DEN alone; however, metformin treatment completedly inhibited the occurence of adenoma and HCC. Hepatic preneoplastic lesions, FCA (Fig 1D) developed in the livers of all mice that received DEN. Metformin treatment significantly reduced the multiplicity of FCA (Fig 1E; *P* < 0.001).

**Table 2:** Incidence and multiplicity of hepatic neoplasms and FCA in the experimental mice.

Group no.	Treatment	No. of mice	Incidence (%)		Multiplicity (no. of neoplasms/mouse) (mean ± SD)					
			Adenoma	HCC	Total tumor		Adenoma		HCC	
1	None	6	0/6 (0)	0/6 (0)	0		0		0	
2	metformin alone	5	0/5 (0)	0/5 (0)	0		0		0	
3	DEN alone	19	6/19 (31.6)	3/19 (15.8)	1.3	± 2.6	0.6	± 1.2	0.6	± 1.6
4	DEN + metformin	13	0/13 (0)^a^	0/13 (0)	0		0		0	

^a^Significantly different from group 3 by Fisher's exact probability test (P < 0.05)

### Effects of metformin on serum levels of glucose and insulin, insulin resistance, and insulin sensitivity

**Fig 2:**
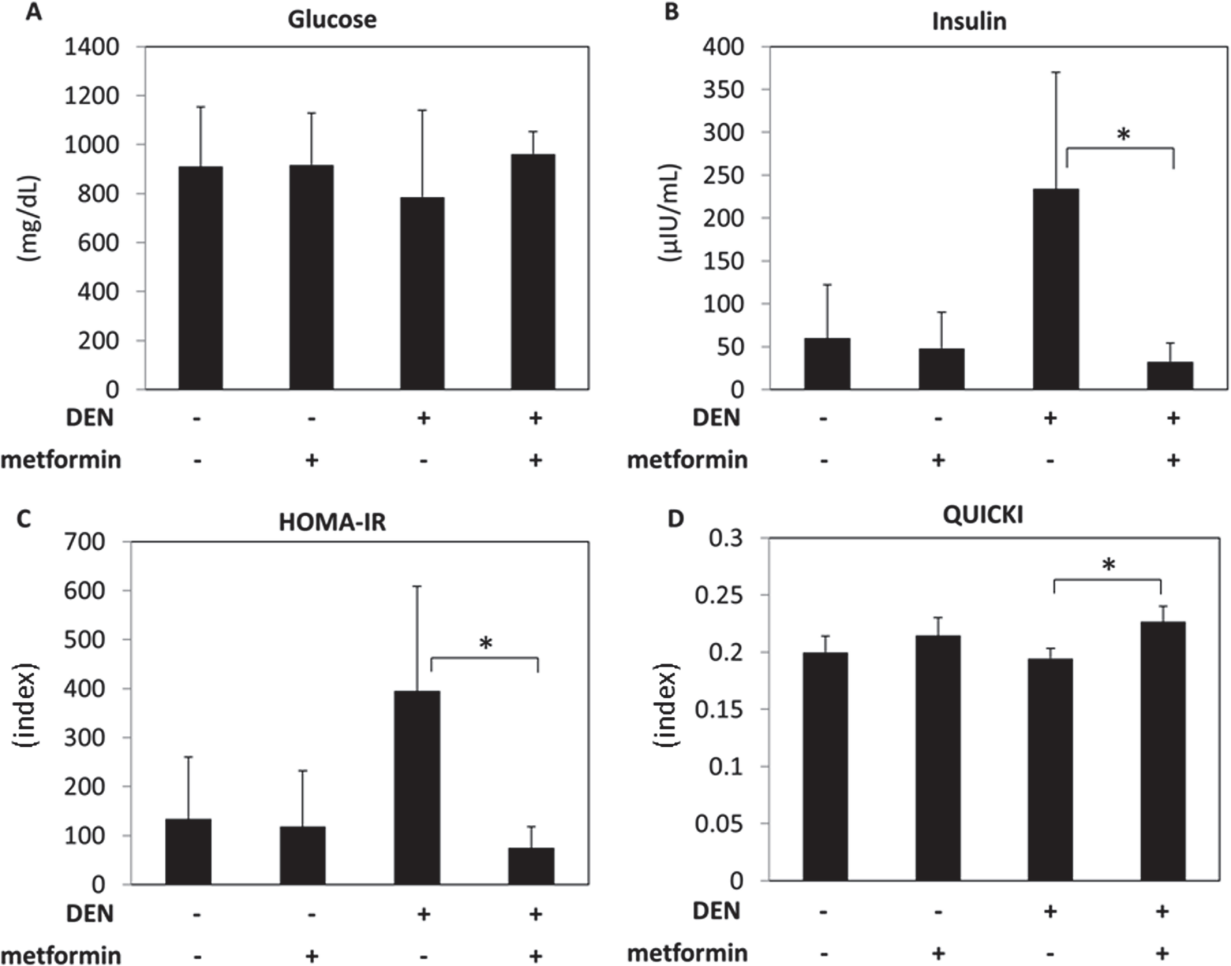
Effect of metformin on serum levels of glucose and insulin, insulin resistance, and insulin sensitivity. Serum levels of glucose (A) and insulin (B) were measured by the mutarotase/glucose oxidase method or enzyme immunoassay, respectively. The values of the HOMA-IR (C) and the QUICKI (D) were calculated to evaluate insulin resistance or insulin sensitivity, respectively. Values are the means ± SD. * *P* < 0.05.

Since insulin resistance plays a critical role in liver carcinogenesis, the effects of metformin treatment on serum levels of glucose and insulin and on improvements in insulin resistance were examined. There were no significant differences in serum glucose levels among groups, irrespective of DEN and metformin treatment (Fig 2A). However, in the DEN-treated groups, administration of metformin markedly decreased serum levels of insulin and HOMA-IR (Fig 2B and 2C; *P* < 0.05). The value of QUICKI, which indicates the degree of insulin sensitivity, was also significantly increased by metformin treatment in DEN-treated mice (Fig 2D; *P* < 0.05).

### Effects of metformin on phosphorylation of Akt, mTOR, p70S6, and AMPK-α in the livers of experimental mice

**Fig 3:**
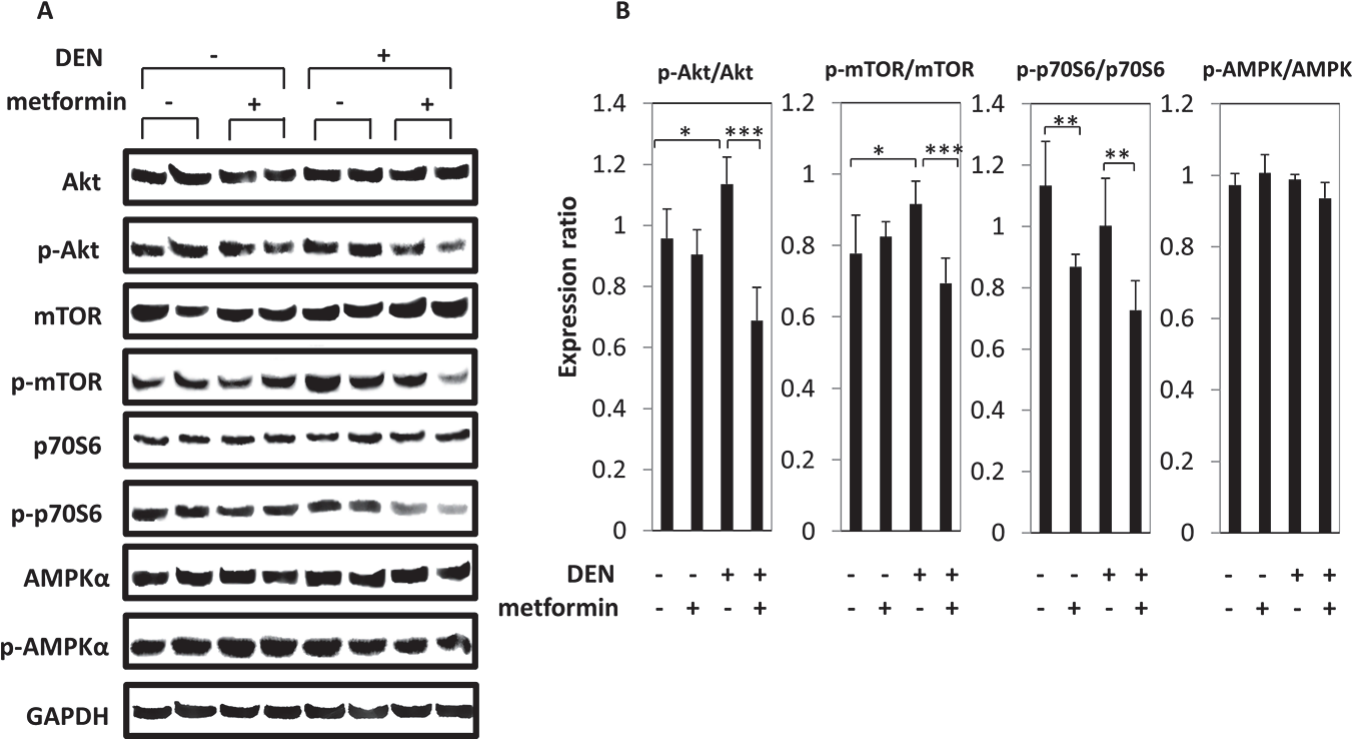
Effect of metformin on phosphorylation of Akt, mTOR, p70S6, and AMPK-α in the liver of experimental mice. (A) Total protein was extracted from the livers of experimental mice and expression of Akt, p-AKT, mTOR, p-mTOR, p70S6, p-p70S6, AMPK-α, and p-AMPK-α was examined by western blot. Repeat western blots gave similar results. (B) Blot intensities were quantified using densitometry. Columns and lines indicate means and SD of triplicate assays. * *P* < 0.05, ** *P* < 0.01, and *** *P* < 0.001.

Activation of the Akt/mTOR pathway has a crucial role in obesity-associated insulin resistance, and is critically important in liver carcinogenesis. Therefore, the effects of metformin treatment on inhibition of Akt, mTOR, and downstream p70S6 phosphorylation in the liver were examined. As shown in Fig 3, western blot analysis revealed that hepatic expression levels of p-Akt and p-mTOR, which were increased by DEN treatment (*P* < 0.05), were significantly decreased by metformin administration (*P* < 0.001). Metformin also reduced hepatic expression of p-p70S6, irrespective of DEN treatment (*P* < 0.01). Levels of p-AMPK-α were unaltered by metformin administration in the present study, regardless of DEN treatment.

### Effects of metformin on serum levels of leptin and adiponectin

**Fig 4:**
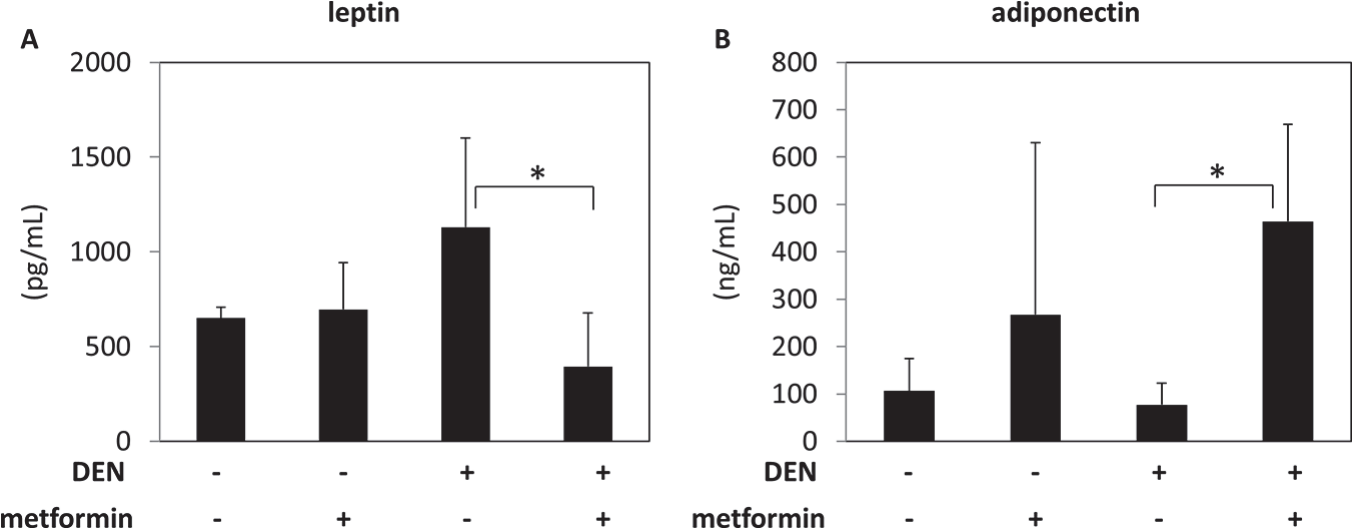
Effect of metformin on serum levels of leptin and adiponectin. Serum levels of leptin (A) and adiponectin (B) were measured by enzyme immunoassay. Values are the means ± SD. * *P* < 0.05.

As adipokine imbalance is associated with liver tumorigenesis, we examined the effects of metformin on serum levels of leptin and adiponectin in the experimental mice. Among the DEN-treated groups, serum leptin levels were decreased significantly by metformin administration (Fig 4A; *P* < 0.05). Conversely, metformin administration significantly increased serum adiponectin levels in DEN-treated *db/db* mice (Fig 4B; *P* < 0.05).

## Discussion

Obesity and diabetes mellitus, which have become more prevalent in the majority of developed countries, are significant risk factors for the development of HCC [2–5]. Reports have suggested that pathophysiological disorders associated with obesity and diabetes, such as insulin resistance and adipokine imbalance, may be promising targets in the prevention of HCC, especially in obese and diabetic patients [8, 9]. The results of the present study show for the first time, that metformin, an anti-diabetic agent used worldwide, effectively prevents the development of DEN-induced liver tumorigenesis in obese and diabetic *db/db* mice probably by ameliorating insulin resistance and adipokine imbalance. Of note, metformin administration completely suppressed development of liver cell neoplasms. Recent clinical reports have also indicated that metformin lowers the risk of several types of cancers, including HCC, in patients with diabetes [23–25]. These clinical findings [23–25], together with the results of the present study, suggest that metformin should be evaluated as a powerful candidate for preventing HCC development in obese individuals, and especially in obese individuals with diabetes.

Recently, a similar effect of metformin has reported by Tajima et al. [36], demonstrating its preventive effect on obesity-related liver tumorigenesis using C57Bl/6 mice fed high-fat diet. The *db/db* mice were selected in the present study because it is a well-known model with high susceptibility to DEN-induced liver tumorigenesis and is thought to be suitable for investigating the development of diabetes- and obesity-related live cancer [17, 20, 42, 43]. Our previous reports have indicated that this high susceptibility might be, at least in part, due to insulin resistance and adipokine imbalance [17, 20, 42, 43]. Another similar literature [22] has shown that metformin prevents DEN-induced liver carcinogenesis in C57BL/6J mice. This is thought interesting since metformin exerts its anti-cancer effects even in lean rodents which appear to have normal state of insulin and adipokine secretions. In our present study, we try to investigate whether metformin is useful to suppress the susceptibility to hepatocarcinogenesis in obese and diabetic mice, which is thought to mimic diabetes- and obesity-related live cancer in human. In addition, metformin is clinically used for diabetic patients, therefore it is considered that clinical trial can be relatively easily conducted in the future with employing not lean but obese and diabetic patients whom metformin is approved to prescribe.

In this study, only female mice were employed. Male rodents are considered to be susceptible to the development of DEN-induced liver cancer compared to female, namely, Naugler et al. [44] have reported that male wild-type mice displayed 100% incidence of DEN-induced liver cancer while females had only 20%. This literature suggested that if we employed male mice in our protocol, the incidence of liver tumor was expected to be too high to compare metformin treated- with untreated-group. In addition, because of the high susceptibility to DEN-induced liver tumorigenesis in *db/db* mice, the tumor incidence would be much higher than that in wild-type mice even for a shorter experimental duration. Therefore, we thought female mice were suitable for the present study.

We considered reduction of serum insulin levels and improvement of insulin sensitivity as one of the most important mechanisms of metformin in the suppression of liver tumorigenesis in obese and diabetic mice, due to the oncogenic properties of insulin on HCC cells, including stimulation of cell growth and anti-apoptotic activity [11–13]. Several animal studies have suggested that targeting higher serum insulin levels and insulin resistance is an effective strategy for inhibiting obesity- and diabetes-related liver tumorigenesis [17, 42, 43]. For instance, dietary supplementation with branched-chain amino acids (BCAA), which improves insulin resistance and glucose tolerance in chronic liver disease patients [45], significantly suppresses liver tumorigenesis in obese and diabetic mice by decreasing serum insulin levels as well as improving insulin sensitivity [17]. Moreover, BCAA supplementation reduced the risk of HCC in obese patients with chronic viral liver disease [46], demonstrating the clinical significance of lowering insulin levels and attenuating insulin resistance in the prevention of liver carcinogenesis in obese and diabetic patients.

It is unclear why serum insulin and leptin levels were higher in DEN-treated *db/db* mice in the present study (Figs 2 and 4). DEN is known as a hepatocarcinogen and has a carcinogenic potency to induce DNA-adducts after being mediated by several enzymes [47, 48]. No literature can be found showing that the serum levels of insulin or leptin differ between DEN-treated and control rodents and that DEN and its metabolite DNA-adduct have direct effects on insulin or leptin levels. According to the previous report, the pro-inflammatory cytokines in chronic hepatitis leads insulin resistance [49], therefore DEN treatment also induced acute and chronic liver injury and might led to production of pro-inflammatory cytokines, resulting in higher serum insulin levels and insulin resistance.

We assessed insulin resistance only by HOMA-IR and QUICKI in this study, since several reports have indicated the validity and reproducibility of those to evaluate insulin resistance [50–52]. Another study, however, suggests that the predictive accuracy of these surrogate indices in mice is not as substantial as those in humans [53]. Therefore, additional studies, such as glucose tolerance, insulin tolerance, and/or euglycemic-hyperinsulinemic clamp tests, should be conducted to confirm whether metformin indeed ameliorates insulin resistance and glucose metabolism in the mouse model. Since the report from Tajima et al. [36] has indicated that metformin restored insulin sensitivity which was examined by insulin tolerance test in mice fed high-fat diet, it is assumed that similar effect of metformin would be observed in the present study. The serum glucose levels were very high (over 800 mg/dl) in accordance with previous reports [17, 54, 55]. Another report [56], however, have shown the glucose level of *db/db* mice can be over 700 mg/dl. There was a possibility that the measuring instrument we use in this study tended to show relatively higher levels of glucose. There was no reports investigating long-term effect of metformin on glucose levels in *db/db* mice, whereas a recent report has shown that short-term administration of metformin does not ameliorate glucose levels in *db/db* mice [57].

Activation of insulin signaling strongly triggers induction of the PI3K/Akt/mTOR pathway in hepatic preneoplastic and neoplastic lesions in rats [14]. Activation of the Akt/mTOR signaling is also involved in HCC development [16]. In the present study, hepatic expression of p-Akt, p-mTOR, and p-p70S6, a protein downstream of mTOR, was significantly decreased by metformin. We presume therefore that metformin inhibits activation of the PI3K/Akt/mTOR pathway, primarily by lowering serum insulin, and that this may suppress liver tumorigenesis in obese and diabetic mice.

In addition to inhibiting activation of the insulin-dependent PI3K/Akt/mTOR pathway, several studies have shown that metformin inhibits mTOR activation through activation of AMPK, which is also a critical mechanism of metformin in exerting its anti-tumor effects [26, 27]. In similar DEN-induced liver carcinogenesis studies using *db/db* mice, due to administration of a synthetic retinoid [42] or a component of green tea catechins [43] the levels of phosphorylated (i.e., activated) AMPK-α were increased, which was considered as one mechanism of suppressing liver tumorigenesis by these agents. In the present study, however, phosphorylated (i.e., activated) AMPK-α was not increased by metformin administration. These findings suggest that AMPK-dependent activity plays a minimal role in suppressing liver tumorigenesis in this mouse study. These findings are also consistent with those of a recent study showing that metformin prevents DEN-induced liver tumorigenesis in mice via AMPK-independent mechanism [22]. It also could be thought that metformin exerted its ameliorating effect on glucose metabolism through activating AMPK rather in muscle than in liver, which might lead to antitumor effect via decreasing serum insulin levels.

Moreover, adipokine imbalance, such as increased serum leptin levels and decreased serum adiponectin levels, is critically involved in obesity- and diabetes-related liver tumorigenesis [17–20], indicating that improving adipokine imbalance may be effective in preventing liver tumorigenesis. The serum leptin levels in DEN-treated group seem to be higher than those in the control group without respective changes in fat mass. The finding appears consistent with a previous study demonstrating that post-hepatitis cirrhotic patients had significantly higher serum leptin levels than healthy control without significant differences in BMI [58]. Although the underling mechanism is still unclear, the differences in serum leptin levels in the present study might be due to chronic liver damage caused by DEN stimulation.

In a previous study, BCAA supplementation effectively suppressed hyperleptinemia and this inhibition was associated with reduced liver carcinogenesis in *db⁄db* mice [17]. Pitavastatin, a lipid-lowering drug, also reduced the development of DEN-induced hepatic preneoplastic lesions in *db⁄db* mice via increasing serum adiponectin levels [20]. Leptin inhibits induction of apoptosis in HCC cells, whereas adiponectin induces apoptosis even in the presence of leptin by inhibiting leptin-induced Akt phosphorylation [19]. Therefore, in the present study, the effects of metformin on amelioration of adipokine imbalance, such as decreasing leptin but increasing adiponectin in serum, may prevent obesity- and diabetes-related liver tumorigenesis. Increased levels of adiponectin in serum and adipose tissue of *db/db* mice due to metformin have also been reported in a previous report [59].

The signaling pathways of leptin and its receptors still remain fully unclear. Among them, the major pathway appears via long-form of leptin receptors (Ob-R_L_) with activating JAK2/STAT, while leptin modulates, directly and indirectly, other signaling pathways, including PI3K and mTOR [60]. The literatures have demonstrated that leptin also functions via JAK2/STAT3 signaling pathway through short-form leptin receptors (Ob-R_S_) [61] and that both Ob-R_L_ and Ob-Rs are expressed in the brain and liver [62, 63], while only Ob-R_L_ is non-functional in *db*/*db* mice. These appear to indicate that leptin also activates STAT3 via Ob-R_S_, which still functions in *db/db* mice. We assumed that leptin could mediate its signaling even in *db/db* mice and, in this study, the difference of serum leptin levels might affect the pathophysiology of the experimental mice, but protein levels of STAT3 and p-STAT3 showed no significant difference among groups (S1 Fig). Since we here focus on insulin and leptin signaling and related protein levels, and the mechanisms of liver carcinogenesis is considered to be complicated, future studies might be needed to address the association of metformin with liver tumorigenesis by investigating other proteins and signaling pathways, and ideally conducting kinome-wide study, related to liver carcinogenesis in addition to insulin and leptin signaling and proteins examined in this study.

Hepatic steatosis, which is often observed in obese individuals and diabetic patients, may also be involved in liver tumorigenesis as hepatic lipid accumulation can induce hepatocyte proliferation and hepatic hyperplasia [64]. Previous studies have also shown that metformin prevents liver tumorigenesis induced by DEN [22] or by a high-fat diet [36] in mice by inhibiting pathways driving hepatic lipogenesis and by reducing fat accumulation in the liver. In the present study, however, improvements by metformin in hepatic steatosis as well as other histopathological findings assessed with NAS were not observed (S2 Fig). We believe this is likely due to the duration of the experiment (20 weeks) as the previous study [36] showing effects of metformin on liver steatosis was a short-term study (8 weeks). Therefore, future studies should be conducted using short-term studies to confirm that metformin reduces hepatic lipid accumulation in this liver tumorigenesis model using *db/db* mice.

In summary, the present study indicates that treatment with the anti-diabetic agent metformin, which has a preventive effect on HCC development based on case-control studies and meta-analyses of human trials [23–25], suppresses DEN-induced liver tumorigenesis in *db/db* mice probably by improving insulin resistance, inhibiting excess activation of the Akt/mTOR signaling, and ameliorating adipokine imbalance. The results of the present study, together with those of previous reports [17, 20, 42, 43], also suggest that targeting obesity- and diabetes-related metabolic abnormalities, including insulin resistance and adipokine imbalance, by either pharmaceutical or nutritional interventions, may be a promising strategy for preventing liver carcinogenesis in obese and diabetic patients who are at an increased risk of HCC development.

## Supporting Information

S1 Fig Effect of metformin on protein levels of STAT3 and phosphorylated STAT3. (TIF)

S2 Fig NAFLD activity score (NAS). (TIF)

## References

[1] The global challenge of diabetes. Lancet. 2008;371:1723 10.1016/S0140-6736(08)60733-3 18502275

[2] Calle EE, Rodriguez C, Walker-Thurmond K, Thun MJ. Overweight, obesity, and mortality from cancer in a prospectively studied cohort of U.S. adults. The New England journal of medicine. 2003;348:1625–38. 12711737 10.1056/NEJMoa021423

[3] Renehan AG, Tyson M, Egger M, Heller RF, Zwahlen M. Body-mass index and incidence of cancer: a systematic review and meta-analysis of prospective observational studies. Lancet. 2008;371:569–78. 10.1016/S0140-6736(08)60269-X 18280327

[4] El-Serag HB, Hampel H, Javadi F. The association between diabetes and hepatocellular carcinoma: a systematic review of epidemiologic evidence. Clinical gastroenterology and hepatology: the official clinical practice journal of the American Gastroenterological Association. 2006;4:369–80. 16527702 10.1016/j.cgh.2005.12.007

[5] Park EJ, Lee JH, Yu GY, He G, Ali SR, Holzer RG, et al Dietary and genetic obesity promote liver inflammation and tumorigenesis by enhancing IL-6 and TNF expression. Cell. 2010;140:197–208. 10.1016/j.cell.2009.12.052 20141834 PMC2836922

[6] Jemal A, Bray F, Center MM, Ferlay J, Ward E, Forman D. Global cancer statistics. CA: a cancer journal for clinicians. 2011;61:69–90. 10.3322/caac.20107 21296855

[7] Imai K, Takai K, Nishigaki Y, Shimizu S, Naiki T, Hayashi H, et al Insulin resistance raises the risk for recurrence of stage I hepatocellular carcinoma after curative radiofrequency ablation in hepatitis C virus-positive patients: A prospective, case series study. Hepatology research: the official journal of the Japan Society of Hepatology. 2010;40:376–82. 10.1111/j.1872-034X.2009.00616.x 20236359

[8] Shimizu M, Kubota M, Tanaka T, Moriwaki H. Nutraceutical approach for preventing obesity-related colorectal and liver carcinogenesis. International journal of molecular sciences. 2012;13:579–95. 10.3390/ijms13010579 22312273 PMC3269707

[9] Shimizu M, Tanaka T, Moriwaki H. Obesity and hepatocellular carcinoma: targeting obesity-related inflammation for chemoprevention of liver carcinogenesis. Seminars in immunopathology. 2013;35:191–202. 10.1007/s00281-012-0336-6 22945457

[10] Pollak M. The insulin and insulin-like growth factor receptor family in neoplasia: an update. Nature reviews Cancer. 2012;12:159–69. 10.1038/nrc3215 22337149

[11] Hagiwara A, Nishiyama M, Ishizaki S. Branched-chain amino acids prevent insulin-induced hepatic tumor cell proliferation by inducing apoptosis through mTORC1 and mTORC2-dependent mechanisms. Journal of cellular physiology. 2012;227:2097–105. 10.1002/jcp.22941 21769869

[12] Kang S, Song J, Kang H, Kim S, Lee Y, Park D. Insulin can block apoptosis by decreasing oxidative stress via phosphatidylinositol 3-kinase- and extracellular signal-regulated protein kinase-dependent signaling pathways in HepG2 cells. European journal of endocrinology / European Federation of Endocrine Societies. 2003;148:147–55. 12534368 10.1530/eje.0.1480147

[13] Tornkvist A, Parpal S, Gustavsson J, Stralfors P. Inhibition of Raf-1 kinase expression abolishes insulin stimulation of DNA synthesis in H4IIE hepatoma cells. The Journal of biological chemistry. 1994;269:13919–21. 8188671

[14] Evert M, Calvisi DF, Evert K, De Murtas V, Gasparetti G, Mattu S, et al V-AKT murine thymoma viral oncogene homolog/mammalian target of rapamycin activation induces a module of metabolic changes contributing to growth in insulin-induced hepatocarcinogenesis. Hepatology. 2012;55:1473–84. 10.1002/hep.25600 22271091

[15] Koketsu Y, Sakoda H, Fujishiro M, Kushiyama A, Fukushima Y, Ono H, et al Hepatic overexpression of a dominant negative form of raptor enhances Akt phosphorylation and restores insulin sensitivity in K/KAy mice. American journal of physiology Endocrinology and metabolism. 2008;294:E719–25. 10.1152/ajpendo.00253.2007 18270303

[16] Calvisi DF, Wang C, Ho C, Ladu S, Lee SA, Mattu S, et al Increased lipogenesis, induced by AKT-mTORC1-RPS6 signaling, promotes development of human hepatocellular carcinoma. Gastroenterology. 2011;140:1071–83. 10.1053/j.gastro.2010.12.006 21147110 PMC3057329

[17] Iwasa J, Shimizu M, Shiraki M, Shirakami Y, Sakai H, Terakura Y, et al Dietary supplementation with branched-chain amino acids suppresses diethylnitrosamine-induced liver tumorigenesis in obese and diabetic C57BL/KsJ-db/db mice. Cancer science. 2010;101:460–7. 10.1111/j.1349-7006.2009.01402.x 19906067 PMC11159020

[18] Kitade M, Yoshiji H, Kojima H, Ikenaka Y, Noguchi R, Kaji K, et al Leptin-mediated neovascularization is a prerequisite for progression of nonalcoholic steatohepatitis in rats. Hepatology. 2006;44:983–91. 17006938 10.1002/hep.21338

[19] Sharma D, Wang J, Fu PP, Sharma S, Nagalingam A, Mells J, et al Adiponectin antagonizes the oncogenic actions of leptin in hepatocellular carcinogenesis. Hepatology. 2010;52:1713–22. 10.1002/hep.23892 20941777 PMC2967627

[20] Shimizu M, Yasuda Y, Sakai H, Kubota M, Terakura D, Baba A, et al Pitavastatin suppresses diethylnitrosamine-induced liver preneoplasms in male C57BL/KsJ-db/db obese mice. BMC cancer. 2011;11:281 10.1186/1471-2407-11-281 21711565 PMC3146939

[21] Stumvoll M, Haring HU, Matthaei S. Metformin. Endocrine research. 2007;32:39–57. 18271504 10.1080/07435800701743828

[22] Bhalla K, Hwang BJ, Dewi RE, Twaddel W, Goloubeva OG, Wong KK, et al Metformin prevents liver tumorigenesis by inhibiting pathways driving hepatic lipogenesis. Cancer prevention research. 2012;5:544–52. 10.1158/1940-6207.CAPR-11-0228 22467080 PMC3324649

[23] Singh S, Singh PP, Singh AG, Murad MH, Sanchez W. Anti-diabetic medications and the risk of hepatocellular cancer: a systematic review and meta-analysis. The American journal of gastroenterology. 2013;108:881–91. 10.1038/ajg.2013.5 23381014

[24] Noto H, Goto A, Tsujimoto T, Noda M. Cancer risk in diabetic patients treated with metformin: a systematic review and meta-analysis. PloS one. 2012;7:e33411 10.1371/journal.pone.0033411 22448244 PMC3308971

[25] Evans JM, Donnelly LA, Emslie-Smith AM, Alessi DR, Morris AD. Metformin and reduced risk of cancer in diabetic patients. BMJ. 2005;330:1304–5. 15849206 10.1136/bmj.38415.708634.F7 PMC558205

[26] Zhou G, Myers R, Li Y, Chen Y, Shen X, Fenyk-Melody J, et al Role of AMP-activated protein kinase in mechanism of metformin action. Journal of Clinical Investigation. 2001;108:1167–74. 11602624 10.1172/JCI13505 PMC209533

[27] Dowling RJ, Zakikhani M, Fantus IG, Pollak M, Sonenberg N. Metformin inhibits mammalian target of rapamycin-dependent translation initiation in breast cancer cells. Cancer research. 2007;67:10804–12. 18006825 10.1158/0008-5472.CAN-07-2310

[28] Larsson O, Morita M, Topisirovic I, Alain T, Blouin MJ, Pollak M, et al Distinct perturbation of the translatome by the antidiabetic drug metformin. Proceedings of the National Academy of Sciences of the United States of America. 2012;109:8977–82. 10.1073/pnas.1201689109 22611195 PMC3384216

[29] Klein J, Westphal S, Kraus D, Meier B, Perwitz N, Ott V, et al Metformin inhibits leptin secretion via a mitogen-activated protein kinase signalling pathway in brown adipocytes. The Journal of endocrinology. 2004;183:299–307. 15531718 10.1677/joe.1.05646

[30] Asensio-Lopez MC, Lax A, Pascual-Figal DA, Valdes M, Sanchez-Mas J. Metformin protects against doxorubicin-induced cardiotoxicity: involvement of the adiponectin cardiac system. Free radical biology & medicine. 2011;51:1861–71. 21907790 10.1016/j.freeradbiomed.2011.08.015

[31] Chen HP, Shieh JJ, Chang CC, Chen TT, Lin JT, Wu MS, et al Metformin decreases hepatocellular carcinoma risk in a dose-dependent manner: population-based and in vitro studies. Gut. 2013;62:606–15. 10.1136/gutjnl-2011-301708 22773548

[32] Cheng J, Huang T, Li Y, Guo Y, Zhu Y, Wang Q, et al AMP-Activated Protein Kinase Suppresses the In Vitro and In Vivo Proliferation of Hepatocellular Carcinoma. PLoS One. 2014;9:e93256 10.1371/journal.pone.0093256 24709998 PMC3977828

[33] Cai X, Hu X, Cai B, Wang Q, Li Y, Tan X, et al Metformin suppresses hepatocellular carcinoma cell growth through induction of cell cycle G1/G0 phase arrest and p21CIP and p27KIP expression and downregulation of cyclin D1 in vitro and in vivo. Oncol Rep. 2013;30:2449–57. 10.3892/or.2013.2718 24008375

[34] Saito T, Chiba T, Yuki K, Zen Y, Oshima M, Koide S, et al Metformin, a diabetes drug, eliminates tumor-initiating hepatocellular carcinoma cells. PLoS One. 2013;8:e70010 10.1371/journal.pone.0070010 23922888 PMC3726625

[35] Toyama K, Nakamura T, Kataoka K, Yasuda O, Fukuda M, Tokutomi Y, et al Telmisartan protects against diabetic vascular complications in a mouse model of obesity and type 2 diabetes, partially through peroxisome proliferator activated receptor-gamma-dependent activity. Biochem Biophys Res Commun. 2011;410:508–13. 10.1016/j.bbrc.2011.06.012 21679694

[36] Tajima K, Nakamura A, Shirakawa J, Togashi Y, Orime K, Sato K, et al Metformin prevents liver tumorigenesis induced by high-fat diet in C57Bl/6 mice. American journal of physiology Endocrinology and metabolism. 2013;305:E987–98. 10.1152/ajpendo.00133.2013 23964070

[37] Popp JA, Goldsworthy TL. Defining foci of cellular alteration in short-term and medium-term rat liver tumor models. Toxicol Pathol. 1989;17:561–8. 2697937 10.1177/0192623389017004102

[38] Frith CH, Ward JM, Turusov VS. Tumours of the liver IARC scientific publications 1994:223–69. 8082908

[39] Kleiner DE, Brunt EM, Van Natta M, Behling C, Contos MJ, Cummings OW, et al Design and validation of a histological scoring system for nonalcoholic fatty liver disease. Hepatology. 2005;41:1313–21. 15915461 10.1002/hep.20701

[40] Ohno T, Shirakami Y, Shimizu M, Kubota M, Sakai H, Yasuda Y, et al Synergistic growth inhibition of human hepatocellular carcinoma cells by acyclic retinoid and GW4064, a farnesoid X receptor ligand. Cancer letters. 2012;323:215–22. 10.1016/j.canlet.2012.04.015 22579649

[41] Katz A, Nambi SS, Mather K, Baron AD, Follmann DA, Sullivan G, et al Quantitative insulin sensitivity check index: a simple, accurate method for assessing insulin sensitivity in humans. J Clin Endocrinol Metab. 2000;85:2402–10. 10902785 10.1210/jcem.85.7.6661

[42] Shimizu M, Sakai H, Shirakami Y, Iwasa J, Yasuda Y, Kubota M, et al Acyclic retinoid inhibits diethylnitrosamine-induced liver tumorigenesis in obese and diabetic C57BLKS/J- +(db)/+Lepr(db) mice. Cancer prevention research. 2011;4:128–36. 10.1158/1940-6207.CAPR-10-0163 21071580

[43] Shimizu M, Sakai H, Shirakami Y, Yasuda Y, Kubota M, Terakura D, et al Preventive effects of (-)-epigallocatechin gallate on diethylnitrosamine-induced liver tumorigenesis in obese and diabetic C57BL/KsJ-db/db Mice. Cancer prevention research. 2011;4:396–403. 10.1158/1940-6207.CAPR-10-0331 21372039

[44] Naugler WE, Sakurai T, Kim S, Maeda S, Kim K, Elsharkawy AM, et al Gender disparity in liver cancer due to sex differences in MyD88-dependent IL-6 production. Science. 2007;317:121–4. 17615358 10.1126/science.1140485

[45] Kawaguchi T, Izumi N, Charlton MR, Sata M. Branched-chain amino acids as pharmacological nutrients in chronic liver disease. Hepatology. 2011;54:1063–70. 10.1002/hep.24412 21563202

[46] Muto Y, Sato S, Watanabe A, Moriwaki H, Suzuki K, Kato A, et al Overweight and obesity increase the risk for liver cancer in patients with liver cirrhosis and long-term oral supplementation with branched-chain amino acid granules inhibits liver carcinogenesis in heavier patients with liver cirrhosis. Hepatology research: the official journal of the Japan Society of Hepatology. 2006;35:204–14. 16737844 10.1016/j.hepres.2006.04.007

[47] Kang JS, Wanibuchi H, Morimura K, Gonzalez FJ, Fukushima S. Role of CYP2E1 in diethylnitrosamine-induced hepatocarcinogenesis in vivo. Cancer Res. 2007;67:11141–6. 18056438 10.1158/0008-5472.CAN-07-1369

[48] Shirakami Y, Gottesman ME, Blaner WS. Diethylnitrosamine-induced hepatocarcinogenesis is suppressed in lecithin:retinol acyltransferase-deficient mice primarily through retinoid actions immediately after carcinogen administration. Carcinogenesis. 2012;33:268–74. 10.1093/carcin/bgr275 22116467 PMC3271263

[49] Cua IH, Hui JM, Bandara P, Kench JG, Farrell GC, McCaughan GW, et al Insulin resistance and liver injury in hepatitis C is not associated with virus-specific changes in adipocytokines. Hepatology. 2007;46:66–73. 17596870 10.1002/hep.21703

[50] Emoto M, Nishizawa Y, Maekawa K, Hiura Y, Kanda H, Kawagishi T, et al Homeostasis model assessment as a clinical index of insulin resistance in type 2 diabetic patients treated with sulfonylureas. Diabetes Care. 1999;22:818–22. 10332688 10.2337/diacare.22.5.818

[51] Sarafidis PA, Lasaridis AN, Nilsson PM, Pikilidou MI, Stafilas PC, Kanaki A, et al Validity and reproducibility of HOMA-IR, 1/HOMA-IR, QUICKI and McAuley's indices in patients with hypertension and type II diabetes. J Hum Hypertens. 2007;21:709–16. 17443211 10.1038/sj.jhh.1002201

[52] Yokoyama H, Emoto M, Fujiwara S, Motoyama K, Morioka T, Komatsu M, et al Quantitative insulin sensitivity check index and the reciprocal index of homeostasis model assessment in normal range weight and moderately obese type 2 diabetic patients. Diabetes Care. 2003;26:2426–32. 12882874 10.2337/diacare.26.8.2426

[53] Lee S, Muniyappa R, Yan X, Chen H, Yue LQ, Hong EG, et al Comparison between surrogate indexes of insulin sensitivity and resistance and hyperinsulinemic euglycemic clamp estimates in mice. Am J Physiol Endocrinol Metab. 2008;294:E261–70. 18003716 10.1152/ajpendo.00676.2007

[54] Minakawa M, Kawano A, Miura Y, Yagasaki K. Hypoglycemic effect of resveratrol in type 2 diabetic model db/db mice and its actions in cultured L6 myotubes and RIN-5F pancreatic beta-cells. J Clin Biochem Nutr. 2011;48:237–44. 10.3164/jcbn.10-119 21562645 PMC3082080

[55] Minakawa M, Miura Y, Yagasaki K. Piceatannol, a resveratrol derivative, promotes glucose uptake through glucose transporter 4 translocation to plasma membrane in L6 myocytes and suppresses blood glucose levels in type 2 diabetic model db/db mice. Biochem Biophys Res Commun. 2012;422:469–75. 10.1016/j.bbrc.2012.05.017 22579688

[56] Cheong SH, Furuhashi K, Ito K, Nagaoka M, Yonezawa T, Miura Y, et al Daidzein promotes glucose uptake through glucose transporter 4 translocation to plasma membrane in L6 myocytes and improves glucose homeostasis in Type 2 diabetic model mice. J Nutr Biochem. 2014;25:136–43. 10.1016/j.jnutbio.2013.09.012 24445037

[57] Eskens BJ, Zuurbier CJ, van Haare J, Vink H, van Teeffelen JW. Effects of two weeks of metformin treatment on whole-body glycocalyx barrier properties in db/db mice. Cardiovascular diabetology. 2013;12:175 10.1186/1475-2840-12-175 24308370 PMC3866460

[58] Bolukbas FF, Bolukbas C, Horoz M, Gumus M, Erdogan M, Zeyrek F, et al Child-Pugh classification dependent alterations in serum leptin levels among cirrhotic patients: a case controlled study. BMC gastroenterology. 2004;4:23 15387890 10.1186/1471-230X-4-23 PMC522814

[59] Fujita H, Fujishima H, Koshimura J, Hosoba M, Yoshioka N, Shimotomai T, et al Effects of antidiabetic treatment with metformin and insulin on serum and adipose tissue adiponectin levels in db/db mice. Endocrine journal. 2005;52:427–33. 16127210 10.1507/endocrj.52.427

[60] Villanueva EC, Myers MG Jr. Leptin receptor signaling and the regulation of mammalian physiology. Int J Obes (Lond). 2008;32 Suppl 7:S8–12. 10.1038/ijo.2008.232 PMC2648306 19136996

[61] Akasaka Y, Tsunoda M, Ogata T, Ide T, Murakami K. Direct evidence for leptin-induced lipid oxidation independent of long-form leptin receptor. Biochim Biophys Acta. 2010;1801:1115–22. 10.1016/j.bbalip.2010.06.009 20601111

[62] Liu ZJ, Bian J, Liu J, Endoh A. Obesity reduced the gene expressions of leptin receptors in hypothalamus and liver. Horm Metab Res. 2007;39:489–94. 17611900 10.1055/s-2007-981680

[63] Sahai A, Malladi P, Pan X, Paul R, Melin-Aldana H, Green RM, et al Obese and diabetic db/db mice develop marked liver fibrosis in a model of nonalcoholic steatohepatitis: role of short-form leptin receptors and osteopontin. American journal of physiology Gastrointestinal and liver physiology. 2004;287:G1035–43. 15256362 10.1152/ajpgi.00199.2004

[64] Yang S, Lin HZ, Hwang J, Chacko VP, Diehl AM. Hepatic hyperplasia in noncirrhotic fatty livers: is obesity-related hepatic steatosis a premalignant condition? Cancer research. 2001;61:5016–23. 11431335

