# Supplementary figures and images for: Metformin Suppresses Diethylnitrosamine-Induced Liver Tumorigenesis in Obese and Diabetic C57BL/KsJ-+Leprdb/+Leprdb Mice
Source: PLoS One. 2015 Apr 16;10(4):e0124081. doi: 10.1371/journal.pone.0124081 (PMC4399835; doi:10.1371/journal.pone.0124081)

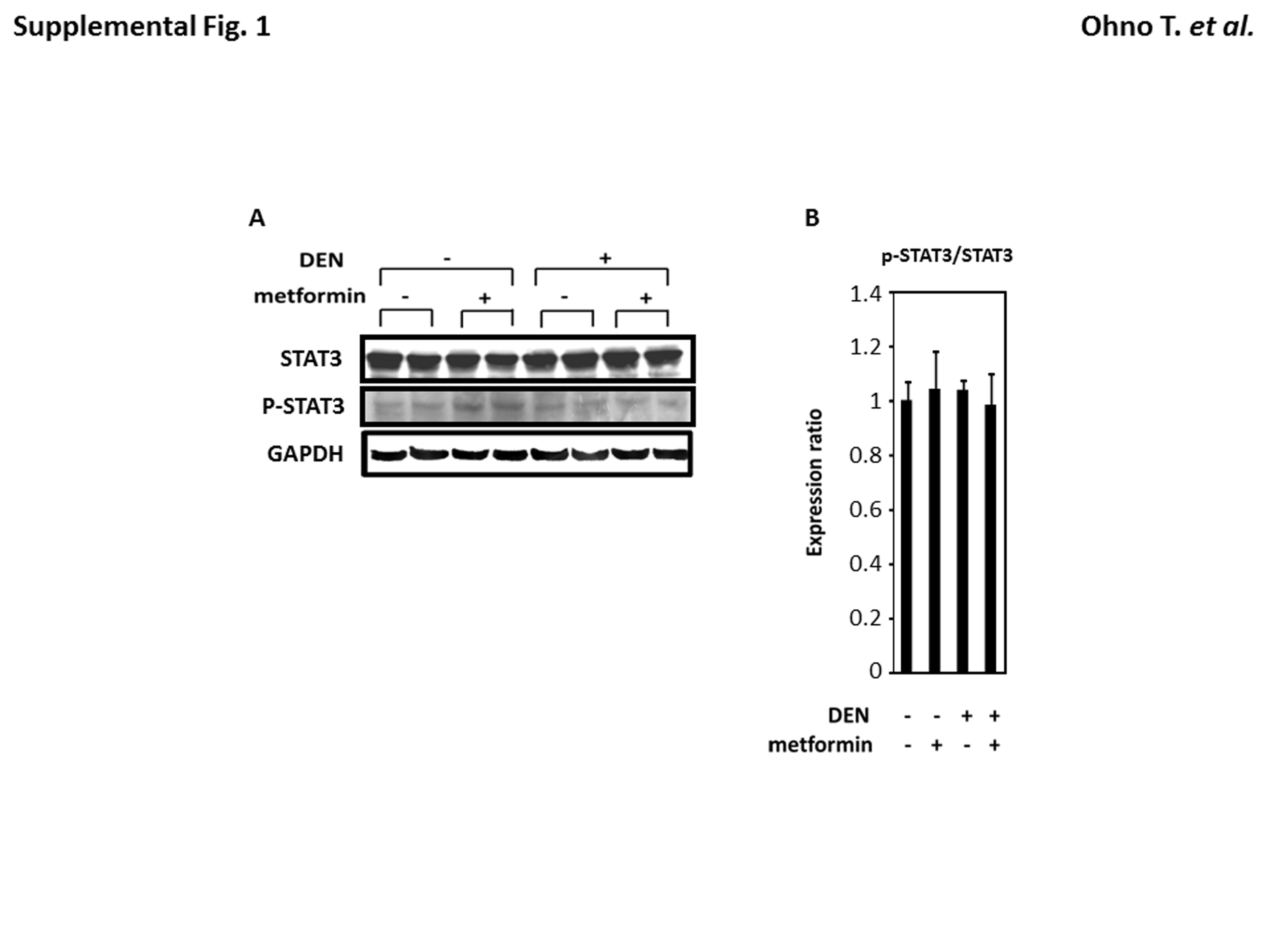

Supplement: S1 Fig — (TIF) [file pone.0124081.s001.tif]

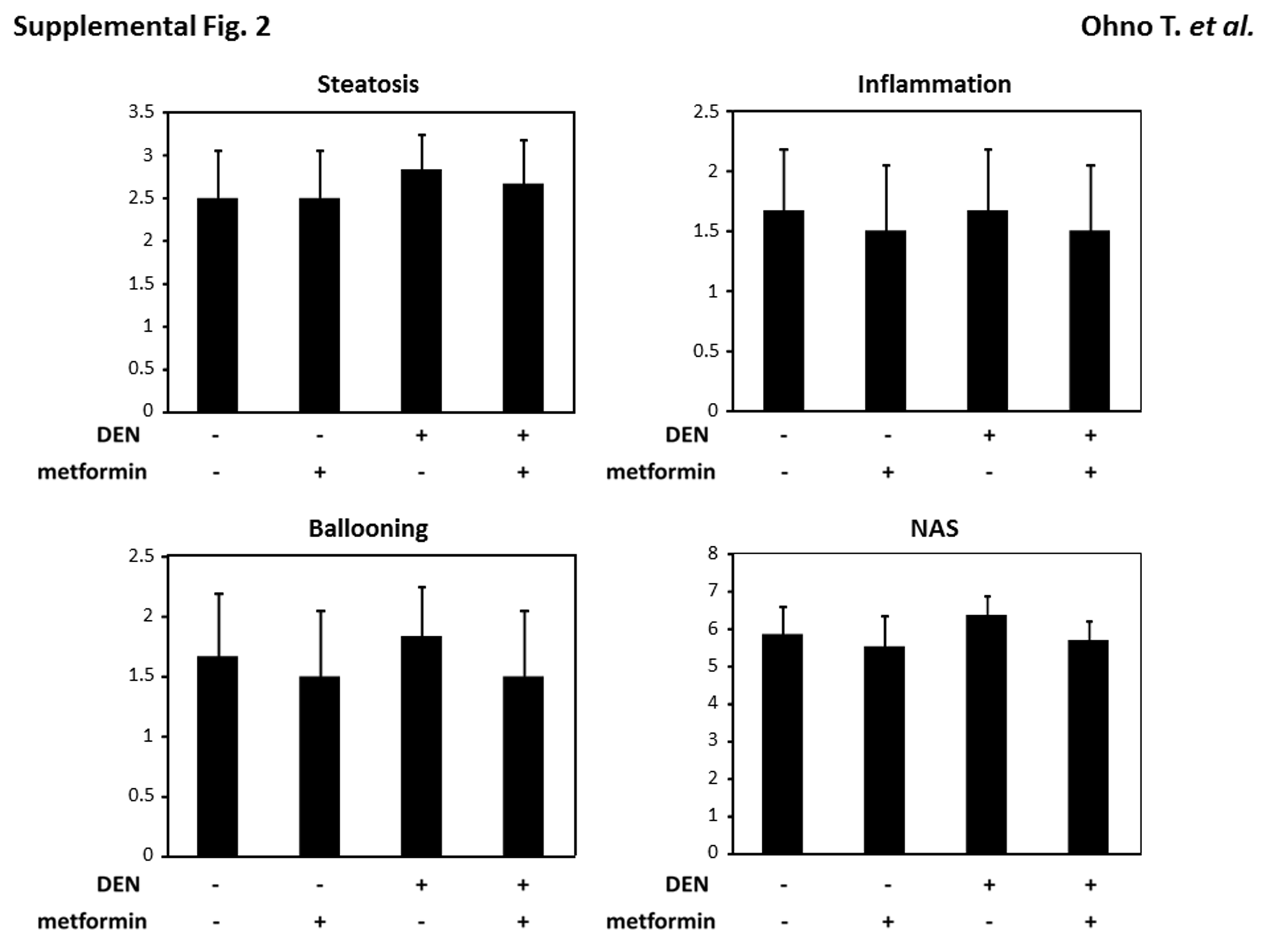

Supplement: S2 Fig — (TIF) [file pone.0124081.s002.tif]
